# Identification of potential key genes involved in iron deficiency for sepsis: A retrospective cohort and transcriptomic study

**DOI:** 10.1097/MD.0000000000050597

**Published:** 2026-09-11

**Authors:** Zehong Wu, Zhangqing Yi, Hanyi Yao, Dongping Li, Haojie Zhou, Jiaming Wu, Yuyang Huang, Weizhi Zhang

**Affiliations:** a Department of Cardiovascular Surgery, The Third Xiangya Hospital of Central South University, Changsha, Hunan, China; b Department of Cardiovascular Surgery, The Second Xiangya Hospital of Central South University, Changsha, Hunan, China; c Clinical Center for Gene Diagnosis and Therapy, the Second Xiangya Hospital of Central South University, Changsha, Hunan, China.

**Keywords:** iron deficiency, Mendelian randomization, MIMIC-IV, sepsis, transcriptomics

## Abstract

Iron overload has been associated with sepsis, but the role of iron deficiency and its molecular links remain unclear. We investigated the association between iron deficiency and sepsis and identified candidate genes potentially linking these conditions. MIMIC-IV data were used to assess the association between serum iron and sepsis status. Transcriptomic datasets from dietary iron-deficient mice (GSE10421), LPS-induced septic mice (GSE267388), and a human blood sepsis cohort (GSE137340) were sequentially analyzed to identify and externally evaluate candidate genes. IEU Open GWAS summary statistics were used for exploratory Mendelian randomization (MR). Exploratory drug prediction was performed using L1000FWD, followed by molecular docking analysis. Patients with sepsis had significantly lower serum iron levels, and restricted cubic spline analysis showed a nonlinear association between serum iron and the odds of sepsis. Cross-tissue transcriptomic analysis identified *Sqle*, *Lss*, and *Rdh11* as candidate genes. In the human blood cohort, SQLE and RDH11 were significantly increased, whereas LSS was not significantly altered. Exploratory MR showed that genetically proxied SQLE expression was associated with higher odds of sepsis (odds ratio [OR] = 1.23, *P* = 1.67 × 10^−3^), whereas LSS expression was associated with lower odds (OR = 0.97, *P* = 8.90 × 10^−4^); RDH11 showed no significant association (OR = 1.01, *P* = .90). Drug prediction identified ML106 as the top-ranked candidate drug, and molecular docking predicted potential binding poses with SQLE and LSS. Serum iron showed a nonlinear association with sepsis status. SQLE, LSS, and RDH11 emerged as candidate genes, with concordant expression changes of SQLE and RDH11 observed in human blood. MR findings for SQLE and LSS were exploratory and require further validation. ML106 was identified through exploratory drug prediction and requires experimental validation before its therapeutic relevance can be established.

## 1. Introduction

Sepsis is a life-threatening syndrome characterized by organ dysfunction resulting from a dysregulated host response to infection.^[1]^ Despite advances in critical care, it remains a major global health burden.^[2]^ In 2017, an estimated 48.9 million cases of sepsis and 11.0 million sepsis-related deaths occurred worldwide, accounting for approximately 19.7% of all deaths.^[3]^ The pathophysiology of sepsis involves complex interactions among inflammation, immune dysfunction, organ injury, and metabolic disturbances.^[4]^ Identifying metabolic factors associated with sepsis may therefore provide additional insight into disease heterogeneity and pathophysiology.

Iron is an essential micronutrient involved in numerous biological processes, including hematopoiesis, cellular respiration, and nucleic acid synthesis and repair.^[5]^ The maintenance of iron homeostasis is critical for health, and its dysregulation has been associated with infections,^[6]^ cardiovascular disorders,^[7]^ immune dysfunction,^[8]^ and neurodegenerative conditions.^[9]^ In the context of infection, iron deficiency (ID) may impair several components of host immune function and has been associated with increased susceptibility to infection. Previous observational studies have also reported associations between iron status and sepsis, including an association between iron overload and increased sepsis risk.^[10]^ However, the relationship between ID and sepsis and the potential molecular links underlying this association remain incompletely understood. Therefore, further investigation of iron status in sepsis may provide insights into the metabolic and molecular alterations associated with the disease.

The Medical Information Mart for Intensive Care IV (MIMIC-IV) is a large-scale, publicly available medical database comprising clinical data from over 50,000 patients collected between 2008 and 2019, including detailed records of surgeries, treatments, and disease outcomes.^[11,12]^ Its extensive sample size and comprehensive clinical metrics make MIMIC-IV a valuable resource for examining clinical associations and potential disease-related factors. For instance, Zheng et al analyzed clinical outcomes, laboratory test results, and comorbidities from 1257 sepsis patients in the MIMIC-IV database, revealing a significant association between an elevated triglyceride-glucose index and increased in-hospital mortality in septic patients.^[13]^ However, while epidemiological studies can reveal associations, they are inherently limited in elucidating underlying molecular mechanisms. Advances in sequencing technologies and bioinformatics have enabled more in-depth mechanistic investigations.^[14]^ Mendelian randomization (MR) uses genetic variants as IVs to strengthen causal inference regarding exposure–outcome relationships under specific instrumental variable assumptions.^[15]^ In the present study, MR was used to provide complementary genetically informed evidence regarding the associations between candidate gene expression and sepsis.

In this study, we combined clinical data from MIMIC-IV with transcriptomic and genetic analyses to examine potential links between iron deficiency and sepsis. Candidate genes identified from iron-deficient and experimental sepsis mouse datasets were subsequently evaluated in an independent human blood cohort. Functional enrichment analysis was used to characterize the biological context of the iron-deficiency-responsive genes, while MR provided complementary genetic evidence. Exploratory drug prediction and molecular docking were then used to prioritize candidate drugs for future experimental study.

## 2. Methods

### 2.1. MIMIC-IV data analysis

#### 2.1.1. Study population

This retrospective study utilized health-related data from the MIMIC-IV database (version 2.2), which contains medical records of patients admitted to the intensive care unit (ICU) at Beth Israel Deaconess Medical Center between 2008 and 2019. Patients aged ≥ 18 years with available measurements of serum iron, transferrin, and total iron-binding capacity (TIBC) within the first 24 hours after ICU admission were included. Patients with missing data for the primary exposure (serum iron) or outcome (sepsis status) were excluded. A total of 18,007 patients met the inclusion criteria.

#### 2.1.2. Definitions

Sepsis was defined according to the Sepsis-3 criteria as suspected or confirmed infection accompanied by an acute increase in the Sequential Organ Failure Assessment (SOFA) score of ≥ 2 points. Patients who did not meet the Sepsis-3 criteria were classified as the non-sepsis group. Iron deficiency was defined according to the laboratory reference ranges recorded in the MIMIC-IV database as serum iron < 45 μg/dL in males and < 30 μg/dL in females. For the primary analysis, iron-related indices were analyzed as continuous variables to characterize their nonlinear associations with sepsis status.

#### 2.1.3. Data extraction

Data extraction was performed using structured query language. The extracted variables included: demographic characteristics, including age, sex, and body mass index (BMI); iron-related laboratory parameters, including serum iron, serum transferrin, and TIBC. Transferrin saturation (%) was calculated using the formula:$\;\;\;Transferrin\;\;\;saturation\;\;\;\left(\;\%\;\right)=\frac{serum\;\;\;iron}{serum\;\;\;TIBC}\times 100\;\%\;$; disease severity at admission, assessed using the SOFA score; and ICU outcomes, including length of stay and in-ICU mortality. Laboratory parameters were based on the first available measurements within the first 24 hours after ICU admission. Additional laboratory variables were extracted for descriptive characterization but were not included as prespecified covariates in the primary RCS models.

#### 2.1.4. Statistical analysis

Continuous variables are presented as median (interquartile range, IQR) and were compared using the Mann–Whitney *U* test. Categorical variables are presented as counts (percentages) and were compared using the chi-square test. For iron-related indices, including serum iron, transferrin, TIBC, and transferrin saturation, between-group differences and their 95% confidence intervals (CIs) were estimated using the Hodges–Lehmann estimator.

To characterize the nonlinear associations between iron-related indices and the odds of sepsis, separate restricted cubic spline (RCS) logistic regression models were fitted for serum iron, transferrin, TIBC, and transferrin saturation using the rms package in R (version 4.4.1). Five knots were placed at the 5th, 25th, 50th, 75th, and 95th percentiles of the distribution of each corresponding iron-related index. The median value of each iron-related index was used as the reference for odds ratio estimation (odds ratio [OR] = 1). All models were adjusted for age, sex, and BMI.

The SOFA score was not additionally included as a covariate because it was used to define sepsis status according to the Sepsis-3 criteria. Although several laboratory variables were available for descriptive characterization, a comprehensive and prespecified set of potential confounders, including comorbidities, transfusion exposure, additional illness-severity measures, and ICU-related characteristics, was not consistently available for multivariable adjustment. Accordingly, the RCS analyses were intended to characterize the shape of the associations between circulating iron-related indices and sepsis status rather than to estimate fully adjusted independent or causal effects. Nonlinearity was assessed by testing the null hypothesis that the coefficients of all nonlinear spline terms were jointly equal to zero. A 2-sided *P*-value < .05 was considered statistically significant.

### 2.2. Transcriptome data analysis

#### 2.2.1. Data acquisition and preprocessing

GSE10421 was included as a murine dataset of hepatic responses to dietary iron deficiency. The dataset contains liver expression profiles from male C57BL/6 and DBA/2 mice maintained on iron-balanced, iron-enriched, or iron-deficient diets, with 5 animals in each strain-by-diet group. For the present differential-expression analysis, only the iron-balanced and iron-deficient groups were included (20 samples in total). Expression profiling was performed using Agilent Whole Mouse Genome 4 × 44 K 1-color microarrays (GPL4134). Expression data downloaded from GEO were processed using norm-exponential background correction with an offset of 50, followed by log_2_ transformation and quantile normalization across arrays.

GSE267388 is a bulk RNA-sequencing dataset of cardiac tissue from a murine model of sepsis-induced myocardial dysfunction. Ten-week-old male wild-type C57BL/6 mice received a single intraperitoneal injection of lipopolysaccharide (LPS; 10 mg/kg) or an equivalent volume of phosphate-buffered saline (PBS). Hearts were collected 12 hours after treatment, with 5 biological replicates in each group. The author-provided gene-level read-count matrix generated on the DNBSEQ-T7 platform was used for downstream analysis. Count data were transformed using the voom function in the limma package to obtain log_2_ counts per million (log_2_ CPM) values with precision weights before linear modeling.

GSE137340 is a human whole-blood microarray dataset generated using the Illumina HumanHT-12 V4.0 Expression BeadChip (GPL10558). The cohort included healthy controls and patients with sepsis sampled at diagnosis (D1, 0 hours) and 24 hours after diagnosis (D2). Based on the sample titles, diagnostic annotations, and collection times, 12 healthy controls, 23 D1 sepsis samples, and 22 D2 sepsis samples were included. Among the patients with sepsis, 19 had matched samples available at both D1 and D2 and were included in the paired longitudinal analysis. The GEO submitters had processed the expression data using the neqc procedure in the limma package, including norm-exponential background correction based on negative-control probes and quantile normalization using negative- and positive-control probes. The resulting author-normalized log_2_-scale expression matrix was used without repeated normalization.

Because the 3 datasets were analyzed independently and were not merged into a single expression matrix, no cross-dataset batch correction was applied.

#### 2.2.2. Differential expression analysis

Differential expression analysis was performed using the limma package (version 3.48.0) in R. For GSE10421, C57BL/6 and DBA/2 mice were analyzed separately by comparing the iron-deficient and iron-balanced groups. For GSE267388, differential expression between LPS-treated and PBS-treated mice was analyzed using the limma-voom framework. For GSE137340, the expression levels of the candidate genes were evaluated in patients with sepsis and healthy controls.

For the genome-wide differential-expression analyses of GSE10421 and GSE267388, genes with an absolute log2 fold change > 0.585, corresponding to a 1.5-fold expression difference, and a Benjamini–Hochberg false discovery rate (FDR) < 0.05 were considered differentially expressed.

#### 2.2.3. Identification of iron-deficiency-related genes

To identify transcriptional changes reproducibly associated with iron deficiency, differential expression analysis was first performed separately in C57BL/6 and DBA/2 mice from GSE10421. Genes meeting the differentially expressed gene (DEG) criteria in both mouse strains and showing concordant directions of change were defined as concordant iron-deficiency-responsive genes and were carried forward for subsequent analyses.

#### 2.2.4. Protein‑protein interaction network and hub gene identification

A protein–protein interaction (PPI) network of the concordant iron-deficiency-responsive genes was constructed using the Search Tool for the Retrieval of Interacting Genes/Proteins (STRING) database. Interactions with a confidence score ≥ 0.4 were retained. The PPI network was visualized using Cytoscape (version 3.10.3), and hub genes were ranked using the maximal clique centrality algorithm implemented in the cytoHubba plugin. The top 10 hub genes were selected for further evaluation.

#### 2.2.5. Functional enrichment analyses

Overrepresentation analysis was performed in R using mouse gene-set annotations distributed with msigdbr version 7.5.1. Gene Ontology (GO) biological process and molecular function, and Kyoto Encyclopedia of Genes and Genomes collections were tested. For each term, enrichment was assessed with a 1-sided hypergeometric test. Terms containing 10 to 500 genes were retained. *P* values were adjusted separately within each database and input gene set using the Benjamini–Hochberg procedure; FDR < 0.05 was considered significant.

#### 2.2.6. Cross-tissue screening and human blood validation

The expression patterns of the top 10 hub genes were subsequently examined in the experimental sepsis dataset GSE267388. Genes showing significant expression changes consistent with the iron-deficiency dataset were considered candidate genes potentially shared between iron deficiency and experimental sepsis. This cross-tissue comparison was used as an exploratory prioritization strategy and was not intended to imply identical transcriptional responses between liver and cardiac tissue.

The candidate genes identified through this procedure were subsequently evaluated in the independent human blood dataset GSE137340. Expression levels were compared between healthy controls and patients with sepsis at diagnosis (D1), with the 24-hour samples used to further examine whether the observed expression patterns persisted over time. Comparisons between healthy controls and D1 or D2 sepsis samples were performed using the 2-sided Wilcoxon rank-sum test. Longitudinal comparisons between D1 and D2 were performed using the 2-sided paired Wilcoxon signed-rank test in the 19 patients with matched samples available at both time points.

### 2.3. Mendelian randomization analysis

#### 2.3.1. Data sources

Genetic variant data for gene-related exposures and sepsis outcomes were obtained from the IEU Open genome-wide association study (GWAS) database.^[16]^ The gene-related variables included SQLE (eqtl-a-ENSG00000104549; 31,470 individuals; 18,881 SNPs), LSS (eqtl-a-ENSG00000160285; 31,684 individuals; 18,265 SNPs), and RDH11 (eqtl-a-ENSG00000072042; 31,644 individuals; 17,798 SNPs). The Ensembl gene IDs were retrieved from the NCBI Gene database. The sepsis outcome data (GWAS summary data, ieu-b-4980) included 11,643 cases of sepsis and 474,841 controls from the UK Biobank.

#### 2.3.2. Instrumental variable selection

The selection of instrumental variables (IVs) was guided by the 3 core assumptions of MR: the genetic variants are strongly associated with the exposure of interest (relevance assumption); the genetic variants are not associated with any confounders of the exposure-outcome relationship (independence assumption); and the genetic variants influence the outcome only through the exposure, not via alternative pathways (exclusion restriction assumption). Single nucleotide polymorphisms (SNPs) associated with each gene-expression exposure at genome-wide significance (*P* < 5 × 10^−8^) were selected as candidate instruments. Linkage disequilibrium (LD) clumping was performed using a 10,000 kb window and an *r*^2^ threshold of < 0.1. Instrument strength was evaluated using the *F*-statistic, and all retained SNPs had *F*-statistics > 10.

#### 2.3.3. Statistical analysis

We employed the inverse-variance weighted (IVW) method as the primary approach to estimate the association between genetically proxied gene expression and the odds of sepsis. To complement the IVW approach, we used the MR-Egger and weighted median methods for additional robustness under varying assumptions regarding pleiotropy. The primary effect measure was the odds ratio (OR) for sepsis, with statistical significance defined as a 2-sided *P*-value < .05.

#### 2.3.4. Sensitivity analysis

Cochran’s *Q* test was used to assess statistical evidence of heterogeneity among SNP-specific estimates, whereas the MR-Egger intercept was used to assess directional horizontal pleiotropy. Leave-one-out analyses were additionally performed to assess whether the overall MR estimates were disproportionately influenced by individual SNPs. A *P*-value > .05 for Cochran’s *Q* or the MR-Egger intercept was interpreted as no statistically significant evidence detected by the corresponding test. All analyses were conducted using the TwoSampleMR package (version 0.5.6) in R (version 4.4.1).

### 2.4. Exploratory drug prediction and molecular docking

#### 2.4.1. Drug screening using L1000 FWD

L1000 Fireworks Display (L1000 FWD)^[17]^ was used for exploratory drug prediction by comparing the candidate gene signature with perturbation-induced transcriptional signatures generated by small molecules. Candidate drugs showing inverse relationships with the input gene-expression signature were ranked according to the metrics provided by L1000FWD, including similarity score, *P*-value, *q* value, *Z* score, and composite score. These metrics were used for computational ranking and prioritization and were not interpreted as evidence of therapeutic efficacy.

#### 2.4.2. Molecular docking

General information on candidate compounds was obtained from PubChem,^[18]^ and 3D structural models were generated using ChemDraw 2022. The corresponding 3-dimensional structures of human target proteins were retrieved from the Protein Data Bank.^[19]^ Protein structures were preprocessed by removing ligands and water molecules using PyMOL (version 3.1.0). Subsequently, protein–small-molecule docking was performed using AutoDock Vina. The predicted ligand–protein binding poses were visualized using PyMOL. Molecular docking was used as an exploratory structural analysis and was not considered evidence of biochemical target engagement, functional modulation, or therapeutic efficacy.

## 3. Results

### 3.1. Clinical characteristics of the MIMIC-IV study population

**Figure 1.**
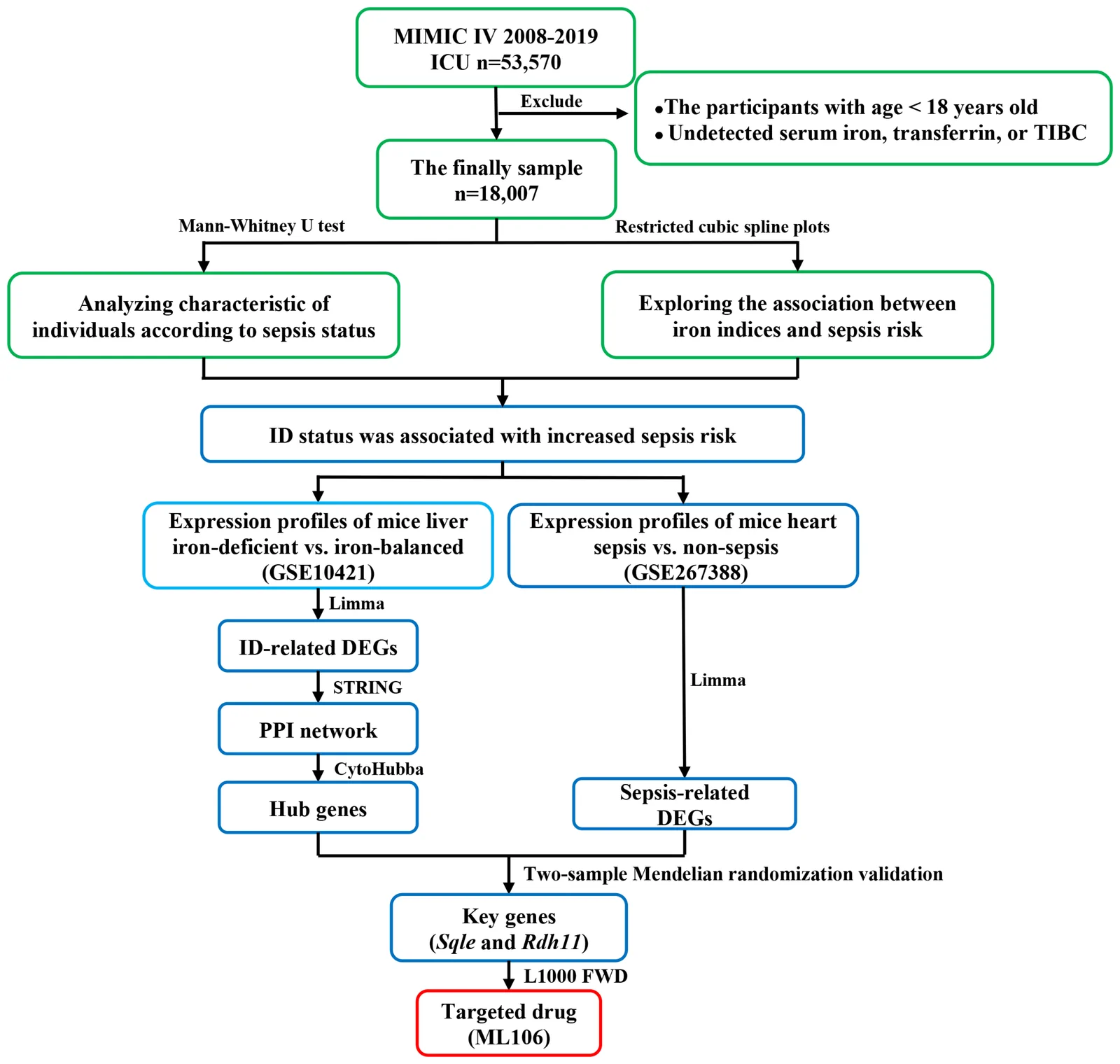
Study workflow and analysis strategy. ICU = intensive care unit, DEG = differentially expressed gene, MIMIC-IV = Medical Information Mart for Intensive Care IV, TIBC = total iron-binding capacity.

**Figure 2.**
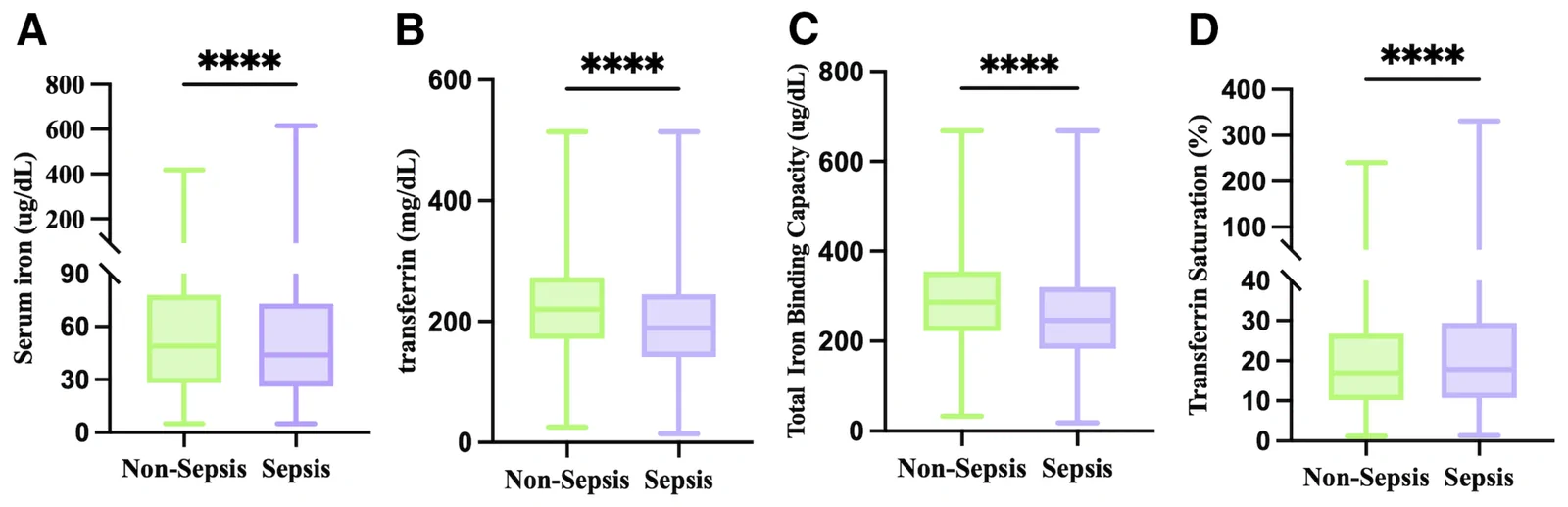
Iron-related indices according to sepsis status. Data were extracted from 18,007 ICU patients (non-sepsis: n = 6804; sepsis: n = 11,203). All laboratory values represent the first recorded measurements within the first 24 hours after ICU admission. Data are presented as median with IQR. Statistical comparisons were performed using the 2-tailed Mann–Whitney *U* test. ****P < .0001 for all comparisons. (A) Serum iron. (B) Serum transferrin. (C) TIBC. (D) Transferrin saturation. ICU = intensive care unit, IQR = interquartile range, TIBC = total iron-binding capacity

The flowchart of the study is presented in Figure 1. Clinical characteristics of the sepsis and non-sepsis groups are summarized in Table 1. As illustrated in Figure 2, compared with patients without sepsis, those with sepsis had lower serum iron (Hodges–Lehmann estimate = −3.00 μg/dL, 95% CI: −4.00 to −2.00, *P* < .001), lower transferrin (−33.00 mg/dL, 95% CI: −35.00 to −30.00, *P* < .001), and lower TIBC (−43.00 μg/dL, 95% CI: −46.00 to −39.00, *P* < .001). In contrast, transferrin saturation was higher in patients with sepsis (1.08%, 95% CI: 0.70 to 1.46, *P* < .001).

**Table 1:** The clinical characteristics of individuals in 2008 to 2019 MIMIC-IV.

Variables*	Non-sepsis (6804)	Sepsis (11203)	*P*
Demographic			
Age	63.00 (51.00, 76.00)	65.00 (54.00, 76.00)	<.001
Gender (male)	3416 (50.21%)	6165 (55.03%)	<.001
BMI (kg/m^2^)	27.80 (24.00, 32.50)	27.70 (23.90, 32.80)	.707
Laboratory tests			
ALT (IU/L)	22.00 (15.00, 37.00)	24.00 (16.00, 43.00)	<.001
AST (IU/L)	25.00 (19.00, 40.00)	29.00 (20.00, 56.00)	<.001
ALP (IU/L)	80.00 (63.00, 108.00)	88.00 (66.00, 126.00)	<.001
GGT (IU/L)	95.00 (35.00, 233.50)	113.00 (46.00, 271.00)	<.001
ALB (g/dL)	3.90 (3.40, 4.30)	3.60 (3.00, 4.10)	<.001
TP (g/dL)	6.90 (6.30, 7.30)	6.70 (6.00, 7.30)	<.001
TBIL (mg/dL)	0.40 (0.30, 0.70)	0.50 (0.30, 1.00)	<.001
BUN (mg/dL)	18.00 (13.00, 26.00)	20.00 (14.00, 20.00)	<.001
SCr (mg/dL)	0.90 (0.80, 1.30)	1.00 (0.80, 1.50)	<.001
TC (mg/dL)	172.00 (140.00, 204.00)	159.00 (128.00, 196.00)	<.001
TG (mg/dL)	117.00 (83.00, 173.00)	122.00 (85.00, 180.00)	<.001
HDL (mg/dL)	49.00 (39.00, 62.00)	46.00 (35.00, 59.00)	<.001
LDL (mg/dL)	91.00 (67.00, 116.00)	83.00 (60.00, 110.00)	<.001
Ca^2+^ (mg/dL)	9.00 (8.50, 9.40)	8.80 (8.20, 9.30)	<.001
Na^+^ (mEq/L)	139.00 (137.00, 141.00)	139.00 (136.00, 141.00)	<.001
K^+^ (mEq/L)	4.20 (3.90, 4.60)	4.20 (3.80, 4.60)	.107
CL^-^ (mEq/L)	102.00 (99.00, 105.00)	102.00 (99.00, 105.00)	.425
HCO_3_^−^ (mEq/L)	25.00 (23.00, 28.00)	25.00 (22.00, 28.00)	<.001
AG (mEq/L)	15.00 (13.00, 17.00)	15.00 (13.00, 17.00)	.025
HB (g/dL)	12.20 (10.50, 13.60)	11.90 (10.20, 13.40)	<.001
WBC (K/uL)	7.80 (6.10, 10.40)	8.20 (6.10, 11.50)	<.001
PLT (K/uL)	242.00 (185.00, 308.00)	225.00 (160.00, 295.00)	<.001
MCV (fL)	90.00 (85.00, 94.00)	91.00 (86.00, 96.00)	<.001
MCH (pg)	30.00 (28.10, 31.60)	30.20 (28.40, 31.90)	<.001
MCHC (g/dL)	33.20 (32.10, 34.20)	33.10 (32.00, 34.10)	.021
RDW (%)	14.10 (13.20, 15.40)	14.50 (13.50, 16.00)	<.001
INR	1.10 (1.00, 1.30)	1.20 (1.10, 1.40)	<.001
PT (s)	12.70 (11.60, 14.20)	13.20 (12.00, 15.50)	<.001
CRP (mg/L)	9.40 (2.40, 53.95)	23.90 (4.50, 81.10)	<.001
Outcomes			
ICU-mortality	2128 (31.28%)	5671 (50.62%)	<.001
ICU-Los-Day	1.43 (0.91, 2.38)	2.66 (1.41, 5.52)	<.001

AG = anion gap, ALB = albumin, ALP = alkaline phosphatase, ALT = alanine aminotransferase, AST = aspartate aminotransferase, BMI = body mass index, BUN = blood urea nitrogen, Ca^2+^ = calcium, Cl^−^ = chloride, CRP (mg/L) = C-reactive protein, GGT = gamma-glutamyl transferase, HB = hemoglobin, HCO_3_^−^ = bicarbonate, HDL = high-density lipoprotein, INR = international normalized ratio, K^+^ = potassium, LDL = low-density lipoprotein, MCH = mean corpuscular hemoglobin, MCHC = mean corpuscular hemoglobin concentration, MCV = mean corpuscular volume, Na^+^ = sodium, PLT = platelet count, PT = prothrombin time, RDW = red cell distribution width, SCr = serum creatinine, TBIL = total bilirubin, TC = total cholesterol, TG = triglycerides, TP = total protein, WBC = white blood cell.

* The complete nomenclature for these variables is provided below.

### 3.2. Associations between iron-related indices and sepsis status in MIMIC-IV

**Figure 3.**
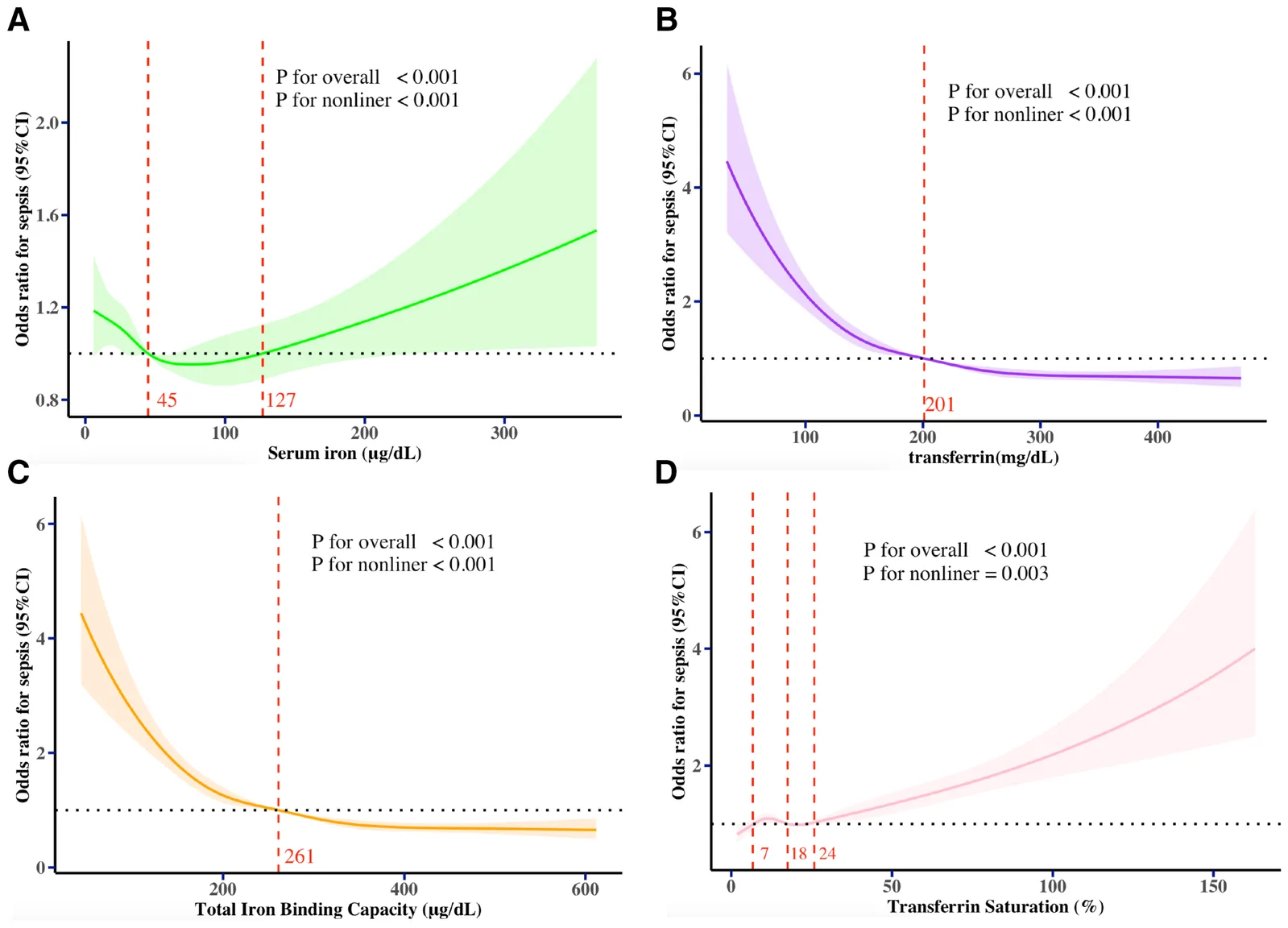
Nonlinear associations between iron-related indices and the odds of sepsis. Odds ratios (solid lines) and 95% CI (shaded areas) were estimated using RCS logistic regression models with 5 knots placed at the 5th, 25th, 50th, 75th, and 95th percentiles of each iron index distribution. Models were adjusted for age, sex, and BMI. (A) Serum iron. (B) Serum transferrin. (C) TIBC. (D) Transferrin saturation. BMI = body mass index, CI = confidence interval, TIBC = total iron-binding capacity.

To characterize the nonlinear associations between iron-related indices and sepsis status, restricted cubic spline logistic regression models adjusted for age, sex, and BMI were fitted in the MIMIC-IV cohort. As illustrated in Figure 3, serum iron exhibited a U-shaped association with the odds of sepsis. Transferrin and TIBC showed L-shaped associations, whereas transferrin saturation showed an N-shaped association. In particular, both relatively low and relatively high serum iron levels were associated with higher odds of sepsis. Given the study focus on iron deficiency and the lower circulating serum iron observed in patients with sepsis, subsequent analyses investigated molecular signatures associated with experimentally induced iron deficiency.

### 3.3. Identification of differentially expressed genes associated with iron deficiency

**Figure 4.**
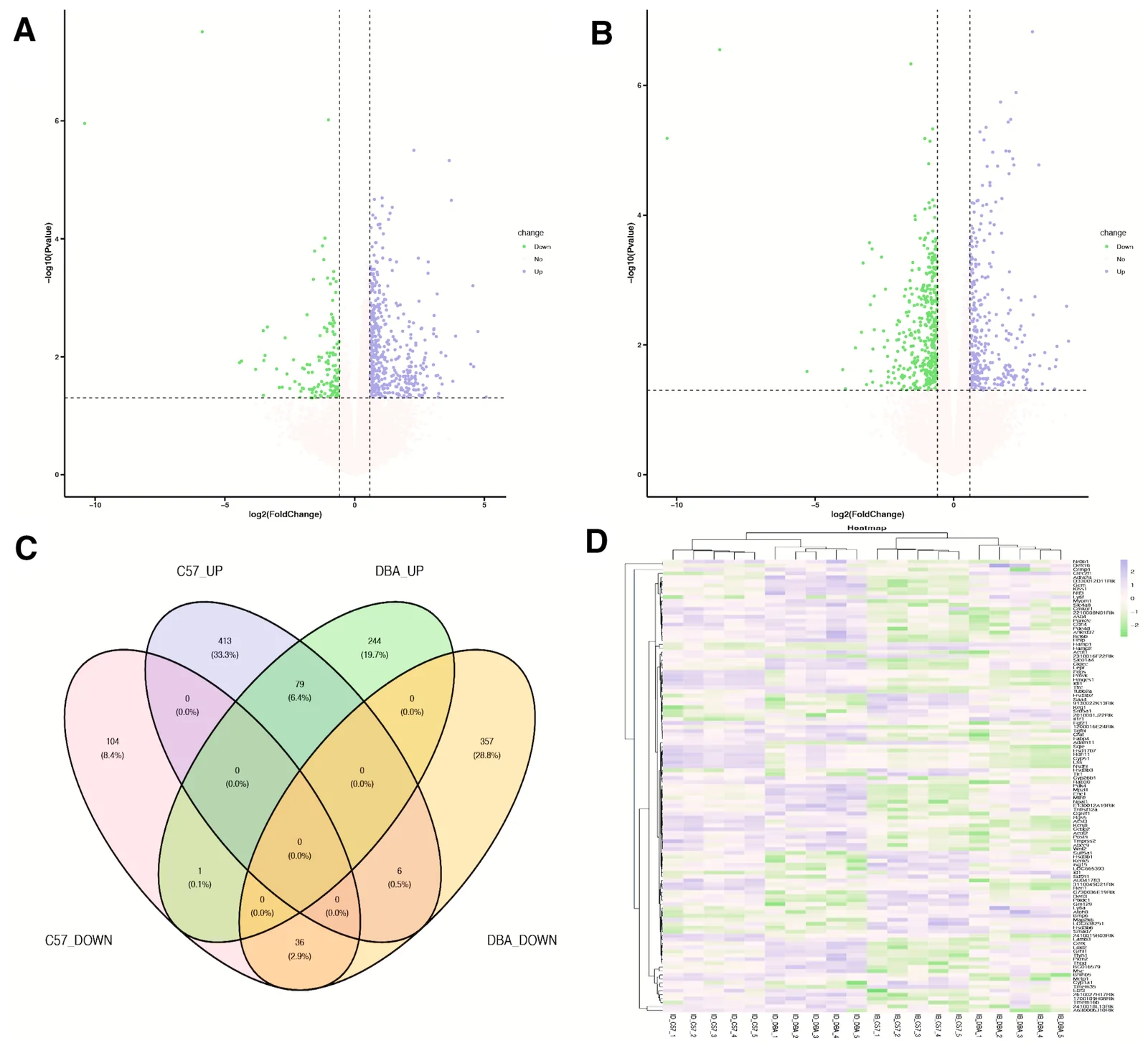
Differential expression analysis of dietary iron deficiency in C57BL/6 and DBA/2 mice. (A) Volcano plot of the differentially expressed genes in C57BL/6 mice. (B) Volcano plot of the differentially expressed genes in DBA/2 mice. (C) Venn diagram of the co-upregulated and co-downregulated DEGs in C57BL/6 and DBA/2 mice. (D) Heatmaps based on differentially expressed genes. DEG = differentially expressed genes.

Using expression profile data obtained from the GEO database, differential gene expression analysis was performed in C57BL/6 and DBA/2 mice subjected to iron-balanced and iron-deficient diets, utilizing the “limma” package in R. Applying a significance threshold of FDR < 0.05 and |log FC| > 0.585, we identified 639 DEGs in C57BL/6 mice (Fig. 4A) and 723 DEGs in DBA/2 mice (Fig. 4B). Due to the distinct genetic backgrounds and biological characteristics of these 2 mouse strains, gene expression changes induced by an iron-deficient diet were complex and exhibited both strain-specific and shared patterns. A Venn diagram (Fig. 4C) revealed 115 concordantly altered DEGs, including 79 concordantly upregulated and 36 concordantly downregulated genes. A heatmap was subsequently generated using a clustering algorithm to visualize the expression patterns of these common DEGs across both strains (Fig. 4D), providing insights into their transcriptional responses to ID.

### 3.4. The protein–protein interaction network of the differentially expressed genes

**Figure 5.**
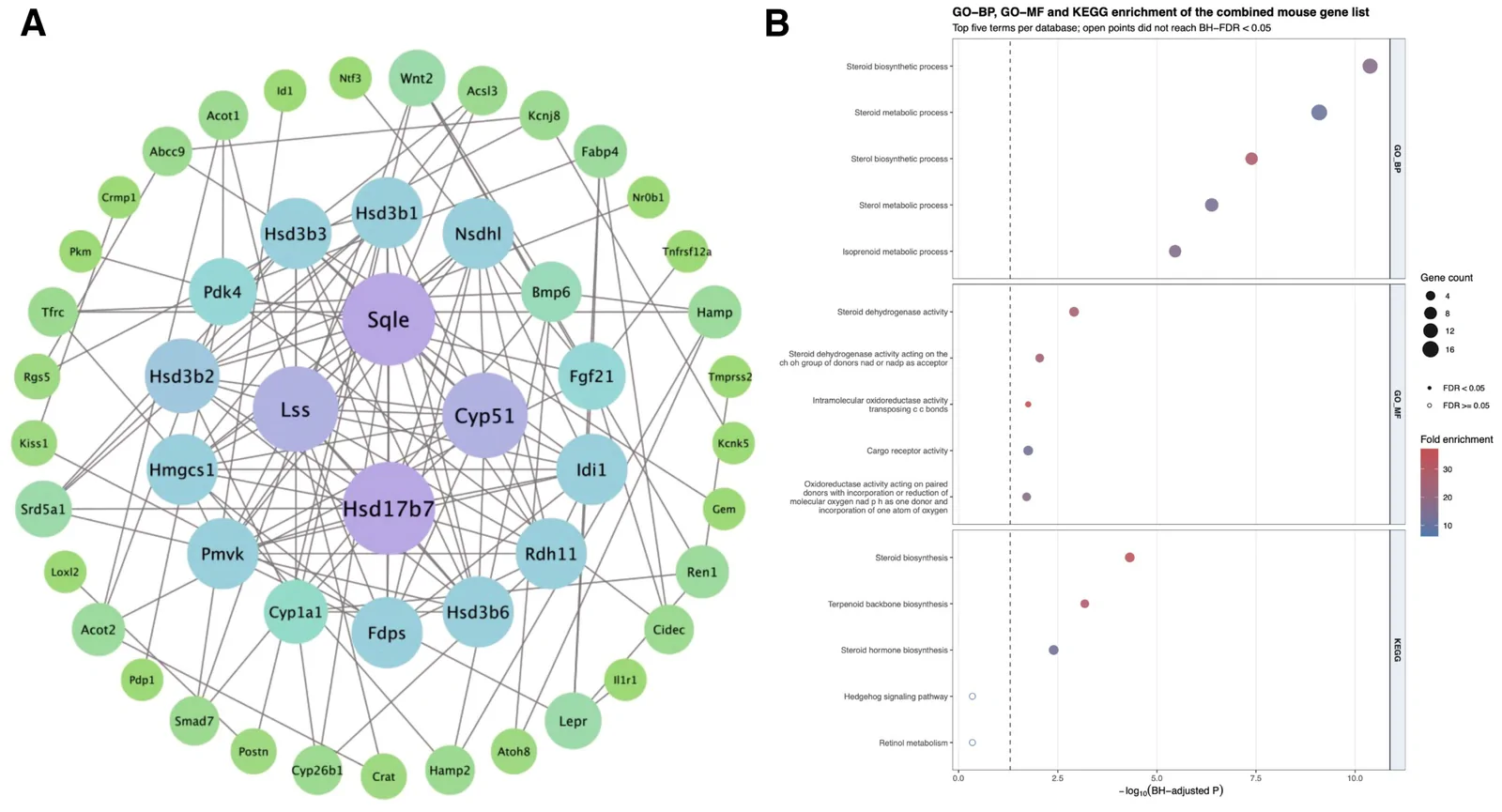
PPI network and functional enrichment analysis of the 115 concordant iron-deficiency-responsive DEGs. (A) Protein–protein interaction (PPI) network of the 115 DEGs shared by C57BL/6 and DBA/2 mice with concordant expression changes. (B) GO biological process (GO-BP), GO molecular function (GO-MF), and KEGG pathway enrichment analyses of these genes. Bubble size represents gene count, and color represents fold enrichment. Filled circles indicate FDR < 0.05. FDR = false discovery rate, GO = Gene Ontology, KEGG = Kyoto Encyclopedia of Genes and Genomes, PPI = protein–protein interaction.

To further explore the interactions among the identified DEGs and uncover potential key genes, a PPI network was constructed using the STRING database. A minimum interaction score threshold of 0.4 was applied, and unconnected nodes were excluded, resulting in a final network comprising 51 interactions. The PPI network was visualized using Cytoscape software (Fig. 5A). To identify hub genes within the network, the cytoHubba plugin was employed, which ranked genes based on maximal clique centrality. Ten hub genes were identified: *Hsd17b7, Sqle, Cyp51, Lss, Pmvk, Fdps, Idi1, Nsdhl, Rdh11*, and *Hmgcs1* (Table 2). These 10 hub genes were selected for subsequent cross-tissue evaluation.

**Table 2:** Differences in the expression of 10 hub genes in C57BL6 and DBA2 mice.

Gene ID	GeneName	C57BL/6		DBA2	
		*P*	Log_2_ FC	*P*	Log_2_ FC
15490	*Hsd17b7*	1.07E−03	1.15	1.80E−06	1.70
20775	*Sqle*	2.24E−02	2.33	1.68E−05	2.18
13121	*Cyp51*	5.42E−03	1.61	3.33E−06	2.06
16987	*Lss*	1.25E−02	0.93	1.07E−05	1.41
68603	*Pmvk*	7.45E−03	0.66	5.89E−05	0.89
110196	*Fdps*	2.34E−02	1.61	1.02E−05	1.89
319554	*Idi1*	9.96E−03	1.72	1.03E−05	2.01
18194	*Nsdhl*	1.32E−03	1.76	3.67E−06	1.97
17252	*Rdh11*	7.86E−03	0.75	6.91E−06	1.10
208715	*Hmgcs1*	6.71E−03	1.15	1.29E−06	2.26

### 3.5. Functional enrichment analysis of iron-deficiency-responsive genes

Functional enrichment analysis was performed on the 115 concordant iron-deficiency-responsive DEGs shared by C57BL/6 and DBA/2 mice (Fig. 5B). GO biological process analysis showed significant enrichment in steroid biosynthetic process, steroid metabolic process, sterol biosynthetic process, sterol metabolic process, and isoprenoid metabolic process. GO molecular function analysis identified enrichment in steroid dehydrogenase activity and several oxidoreductase-related activities. Kyoto Encyclopedia of Genes and Genomes analysis further revealed significant enrichment in steroid biosynthesis, terpenoid backbone biosynthesis, and steroid hormone biosynthesis. These enrichment patterns highlight sterol- and steroid-related metabolism as a prominent transcriptional feature of dietary iron deficiency.

### 3.6. Cross-tissue identification of candidate genes associated with iron deficiency and experimental sepsis

**Figure 6.**
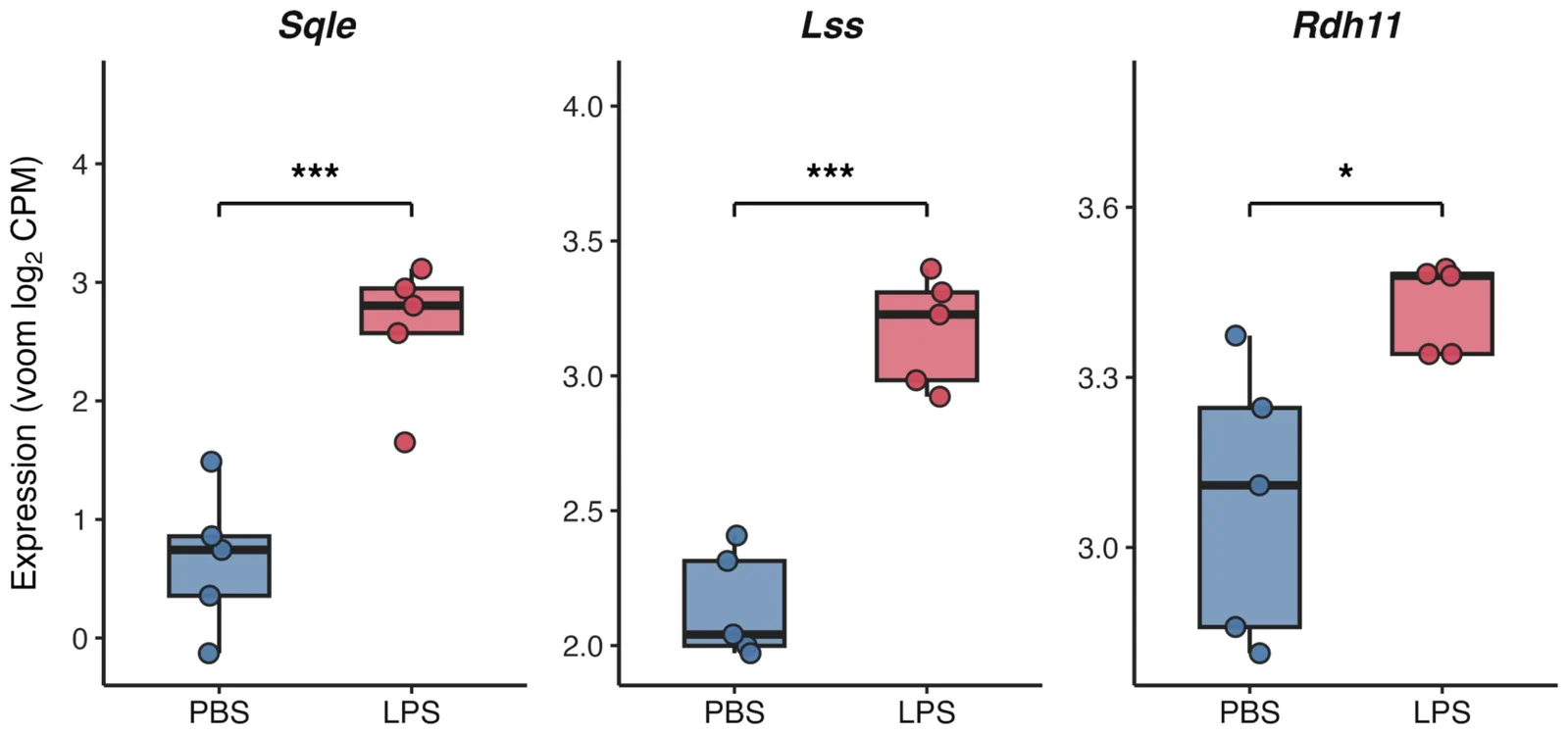
Expression of *Sqle, Lss, and Rdh11* in the experimental sepsis dataset GSE267388. Gene expression was compared between PBS-treated control mice and LPS-treated mice (n = 5 per group). Expression values are shown as voom-transformed log_2_ counts per million (log_2_ CPM). Boxes represent the interquartile range, horizontal lines indicate the median, and individual points represent biological samples. Statistical significance was assessed using the limma-voom framework. *FDR < 0.05, ***FDR < 0.001. FDR = false discovery rate, LPS = lipopolysaccharide, PBS = phosphate-buffered saline, RDH11 = retinol dehydrogenase 11.

The top hub genes derived from the iron-deficiency liver transcriptome were subsequently evaluated in the GSE267388 mouse cardiac transcriptome to identify transcriptional signals shared with experimental sepsis. Three genes *(Sqle, Lss,* and *Rdh11*) were significantly upregulated in LPS-treated mice compared with PBS-treated controls (Fig. 6). These findings identified *Sqle, Lss,* and *Rdh11* as candidate genes for further validation rather than establishing tissue-independent regulation.

### 3.7. External validation of candidate genes in a human blood sepsis cohort

**Figure 7.**
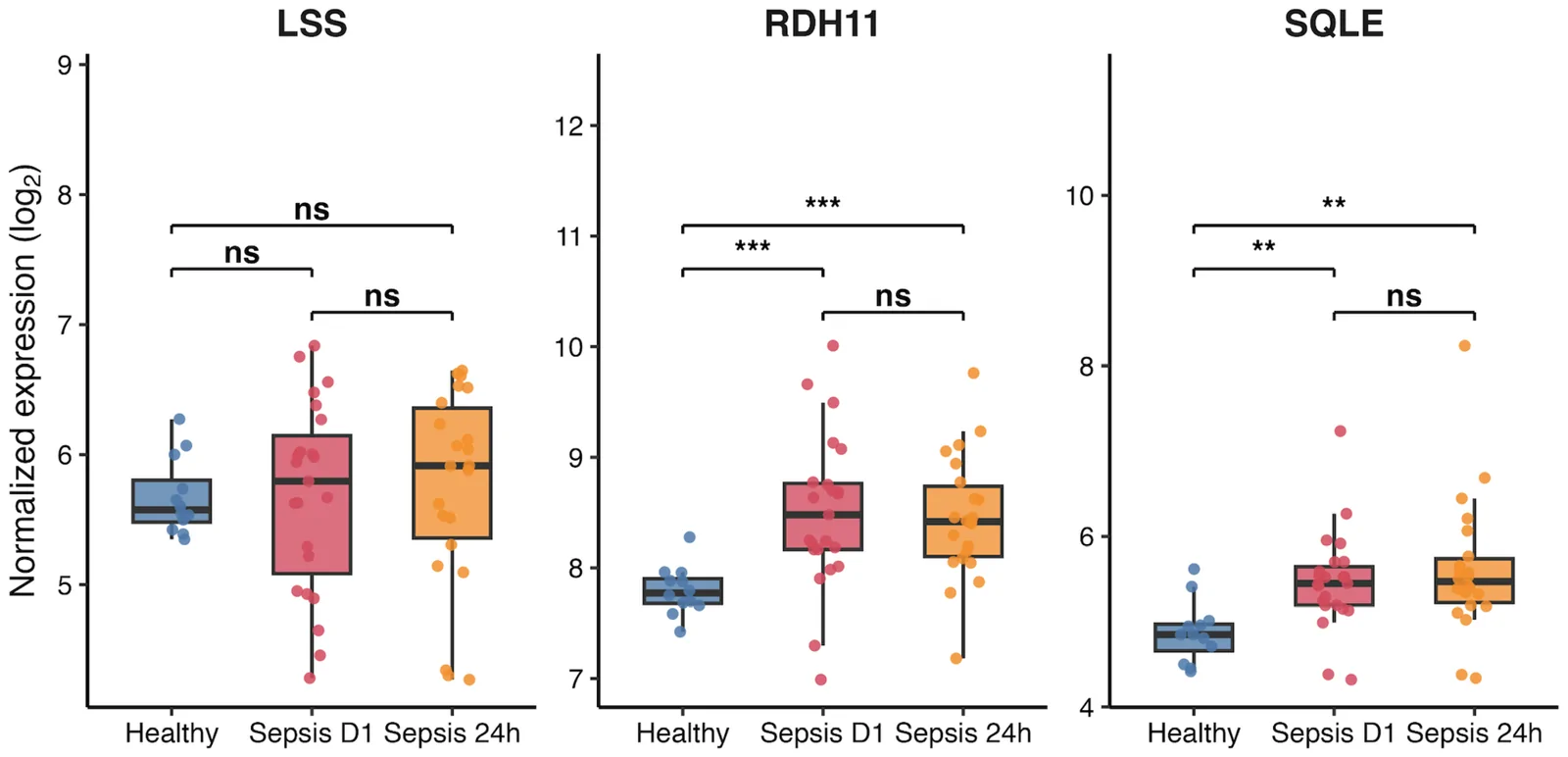
External validation of SQLE, LSS, and RDH11 expression in the human blood sepsis dataset GSE137340. Expression levels were evaluated in healthy controls, patients with sepsis at diagnosis (D1), and samples collected 24 hours after diagnosis (D2). Comparisons between healthy controls and D1 or D2 sepsis samples were performed using the 2-sided Wilcoxon rank-sum test. Longitudinal comparisons between D1 and D2 were performed using the 2-sided paired Wilcoxon signed-rank test in 19 patients with matched samples at both time points. Boxes indicate the median and interquartile range, and individual points represent individual samples. ns, not significant, **P < .01, ***P < .001. LSS = lanosterol synthase, RDH11 = retinol dehydrogenase 1, SQLE = squalene epoxidase.

To assess whether the candidate genes identified in the mouse cross-tissue analysis were also altered in human sepsis, we examined their expression in the independent blood transcriptomic dataset GSE137340. Compared with healthy controls, SQLE and RDH11 expression levels were significantly increased at the time of sepsis diagnosis (D1) and remained elevated 24 hours later (Fig. 7). In contrast, LSS expression did not differ significantly between healthy controls and either sepsis time point. Among the 19 patients with paired samples, none of the 3 candidate genes showed a significant change between D1 and 24 hours. These findings provide human blood-level support for SQLE and RDH11, whereas the transcriptional alteration of LSS observed in mouse cardiac tissue was not reproduced in this human cohort.

### 3.8. Exploratory Mendelian randomization analysis of candidate gene expression and sepsis

We conducted a 2-sample MR analysis using eQTL data for the 3 candidate genes and GWAS summary statistics for sepsis derived from the GWAS database. Using the prespecified instrument-selection criteria (*P* < 5 × 10^−8^; clump_kb = 10,000; LD r^2^ < 0.1), 6 SNPs for SQLE, 36 SNPs for LSS, and 3 SNPs for RDH11 were retained as genetic instruments. These SNPs were used as IVs for the exposures, with sepsis as the outcome. The analysis was performed using the TwoSampleMR package in R, and the results are summarized in Table 3.

**Table 3:** Mendelian randomization estimates of the association between ID-related genes and sepsis risk.

Gene	SNP (n)	Method	*P*	OR
*Sqle*	6	IVW	1.67E−03	1.23 (1.08, 1.40)
		MR Egger	.21	1.55 (0.86, 2.79)
		Weighted median	.02	1.20 (1.02, 1.41)
*Lss*	36	IVW	8.90E−04	0.97 (0.95, 0.99)
		MR Egger	.43	0.99 (0.95, 1.02)
		Weighted median	.10	0.98 (0.95, 1.01)
*Rdh11*	3	IVW	.90	1.01 (0.88, 1.15)
		MR Egger	.94	0.98 (0.67, 1.43)
		Weighted median	.98	1.00 (0.87, 1.15)

IVW = inverse-variance weighted, OR = odds ratio, SNP = single nucleotide polymorphisms.

**Figure 8.**
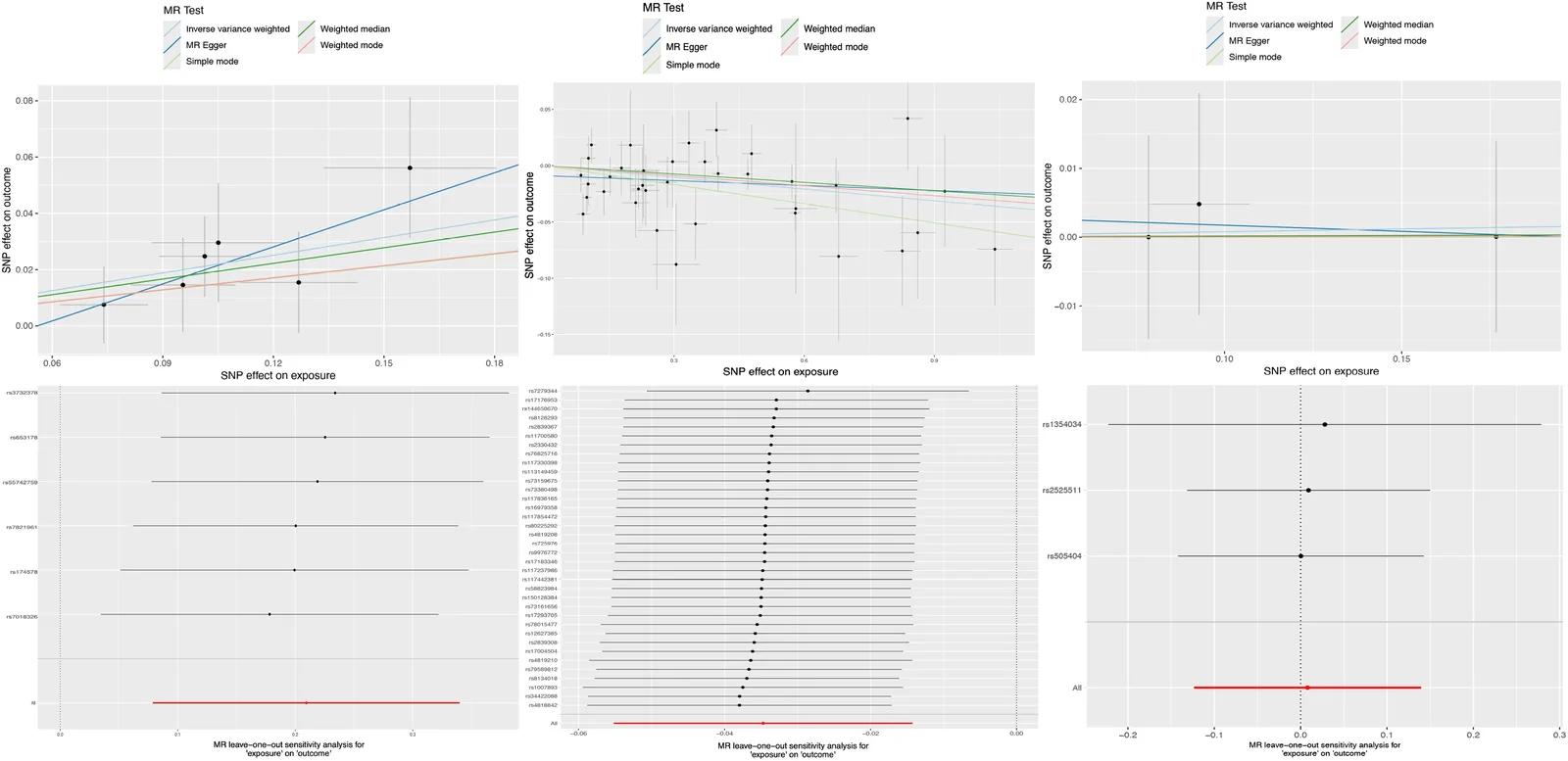
Exploratory MR analyses of candidate gene expression and sepsis. (A–C) Scatter plots showing the associations between genetically proxied SQLE, LSS, and RDH11 expression and sepsis using different MR methods. (D–F) Leave-one-out sensitivity analyses for SQLE, LSS, and RDH11, respectively, evaluating the influence of individual SNPs on the overall MR estimates. LSS = lanosterol synthase, MR = Mendelian randomization, RDH11 = retinol dehydrogenase 11, SNP = single nucleotide polymorphisms, SQLE = squalene epoxidase.

The IVW analysis showed that genetically proxied SQLE expression was associated with higher odds of sepsis (OR = 1.23, *P* = 1.67 × 10^−3^). Genetically proxied LSS expression was associated with lower odds of sepsis (OR = 0.97, *P* = 8.90 × 10^−4^), whereas no statistically significant association was observed for RDH11 (OR = 1.01, *P* = .90). These findings are illustrated in Figure 8A–C, where scatter plots show the estimated associations obtained using different MR methods. Leave-one-out analyses (Fig. 8D–F) were additionally performed to assess the influence of individual SNPs on the overall MR estimates. Cochran’s *Q* and MR-Egger intercept tests did not detect statistically significant heterogeneity or directional pleiotropy. The results of MR-Egger intercept tests and heterogeneity analyses are provided in Tables S1 and S2, Supplemental Digital Content 1, respectively. However, these findings should be interpreted cautiously because the limited number of instruments, particularly for SQLE and RDH11, reduces the power of these diagnostic tests.

### 3.9. Exploratory drug prediction and molecular docking

Using L1000FWD, we identified and ranked the top 10 candidate drugs whose perturbational transcriptional signatures showed inverse relationships with the candidate gene signature (Table 4). BRD-K70693222 (ML106) ranked first and was therefore selected for subsequent exploratory molecular docking analysis.

**Table 4:** Candidate drugs of sepsis in the L1000 FWD.

Drug	Similar Score	*P*-value	*q* value	*Z* score	Combined score
BRD-K70693222	−0.5	3.17E−02	2.05E−01	1.83	−2.74
curcumin	−0.5	3.15E−02	2.05E−01	1.81	−2.72
BRD-K89059493	−0.5	3.28E−02	2.05E−01	1.81	−2.68
RO-28-1675	−0.5	3.04E−02	2.05E−01	1.75	−2.66
BRD-A06131576	−0.5	3.11E−02	2.05E−01	1.75	−2.64
phenolphthalein	−0.5	3.31E−02	2.05E−01	1.76	−2.6
FU-JMBII127B	−0.5	3.51E−02	2.05E−01	1.76	−2.56
BRD-K37940862	−0.5	3.59E−02	2.05E−01	1.75	−2.52
fluticasone	−0.5	3.62E−02	2.05E−01	1.73	−2.5
BRD-K24426149	−0.5	3.58E−02	2.05E−01	1.72	−2.49

**Figure 9.**
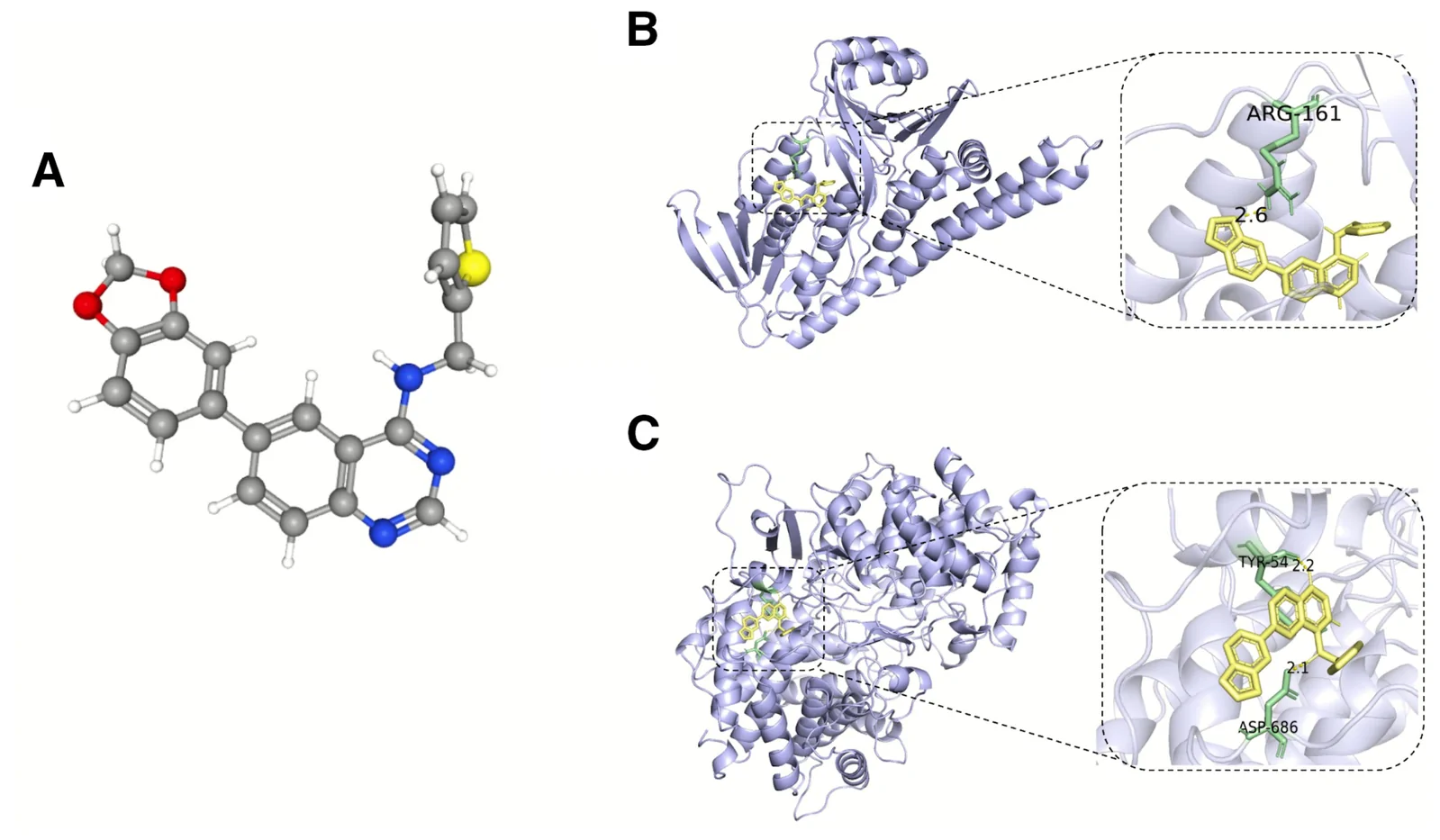
3D structure visualization of ML106 and molecular docking results. (A) Chemical structure of ML106. (B) Predicted binding mode of ML106 to SQLE. A hydrogen bond with ARG-161 is shown as a yellow dashed line (distance: 2.6 Å). (C) Predicted binding mode of ML106 to LSS. Hydrogen bonds with TYR-54 and ASP-686 are shown as yellow dashed lines (distances: 2.2 and 2.1 Å). Docking scores were −7.7 kcal/mol for SQLE and −7.4 kcal/mol for LSS. LSS = lanosterol synthase, SQLE = squalene epoxidase.

ML106 (PubChem CID: 3232621) has the molecular formula C_20_H_15_N_3_O_2_S and a molecular weight of 361.4 g/mol. Its chemical structure is shown in Figure 9A. Molecular docking was subsequently performed using SQLE (PDB ID: 6C6P) and LSS (PDB ID: 1W6J) as target protein structures. ML106 showed a predicted hydrogen-bond interaction with ARG161 of SQLE at 2.6 Å (Fig. 9B) and predicted hydrogen-bond interactions with TYR54 and ASP686 of LSS at 2.2 Å and 2.1 Å, respectively (Fig. 9C). The AutoDock Vina docking scores were −7.7 kcal/mol for SQLE and−7.4 kcal/mol for LSS. These results indicate computationally predicted interactions between ML106 and the selected protein structures but do not establish biochemical binding, target engagement, or functional modulation.

## 4. Discussion

In the MIMIC-IV cohort, circulating iron-related indices showed nonlinear associations with sepsis status. In particular, both relatively low and relatively high serum iron levels were associated with higher odds of sepsis after adjustment for age, sex, and BMI. Previous studies have also highlighted the high prevalence and diagnostic complexity of iron deficiency in patients with sepsis, particularly because systemic inflammation substantially alters conventional iron-status biomarkers.^[20]^ Several mechanisms may underlie this association. Iron deficiency can impair immune function, whereas systemic inflammation during sepsis can itself lower circulating iron through inflammation-mediated iron sequestration.^[21]^ Thus, low serum iron in patients with sepsis may reflect both preexisting iron deficiency and acute inflammatory redistribution, complicating causal interpretation.

Building upon the clinical observations, we used a cross-tissue transcriptomic strategy to explore potential molecular links between iron deficiency and sepsis. GSE10421 served as the discovery dataset for transcriptional responses to dietary iron deficiency, whereas GSE267388 was used to screen these signals in an experimental mouse model of sepsis. This strategy was intended to prioritize potentially shared molecular responses rather than to assume equivalent transcriptional regulation between liver and heart. Three candidate genes, *Sqle, Lss,* and *Rdh11*, were upregulated in the mouse sepsis dataset. Importantly, external evaluation in the independent human blood dataset GSE137340 demonstrated increased SQLE and RDH11 expression in patients with sepsis at diagnosis and 24 hours later, whereas LSS was not significantly altered. These findings indicate partial rather than complete cross-species and cross-tissue replication and suggest that SQLE and RDH11 exhibit the most consistent transcriptional alterations across the experimental and human datasets.

MR analysis provided complementary genetically informed evidence for associations of SQLE and LSS expression with sepsis. Genetically proxied SQLE expression was associated with higher odds of sepsis, whereas genetically proxied LSS expression was associated with lower odds of sepsis. However, these findings should be interpreted cautiously because the number of available genetic instruments was limited, particularly for SQLE and RDH11, and the LD clumping threshold used in the present analysis was relatively liberal. Therefore, the MR findings should be regarded as supportive, hypothesis-generating genetic evidence rather than definitive proof that altered SQLE or LSS expression causally influences sepsis.

SQLE encodes squalene epoxidase, a key enzyme in the cholesterol biosynthesis pathway, whereas LSS encodes lanosterol synthase, which catalyzes another essential step in sterol synthesis.^[22-24]^ In the present study, the enrichment of sterol- and steroid-related processes in the iron-deficiency dataset, together with the altered expression of SQLE and LSS, suggests that sterol metabolism may be involved in the metabolic response to iron deficiency. Cholesterol metabolism is closely linked to membrane organization, lipid-raft-dependent signaling, and immune-cell function.^[23,25-27]^ More broadly, sepsis is accompanied by substantial immunometabolic and lipid remodeling, and disturbances in cholesterol homeostasis may influence inflammatory signaling, host defense, and immune-cell responses.^[26]^ Importantly, the coordinated transcriptional changes in cholesterol biosynthesis-related genes observed in our study should not be interpreted as evidence that enhanced cholesterol synthesis directly drives sepsis. These alterations may instead reflect adaptive responses to inflammatory stress or secondary metabolic remodeling. The divergent MR associations observed for SQLE and LSS, despite their participation in the same metabolic pathway, further suggest that individual genes within the sterol biosynthesis pathway may have distinct and context-dependent biological effects. Therefore, we interpret the observed sterol-related transcriptional signature as part of broader metabolic remodeling potentially linking iron deficiency and sepsis, rather than as an established causal pathway. Further experimental studies are needed to determine the cell-specific and functional roles of SQLE and LSS in this context.

Finally, L1000FWD analysis prioritized ML106 based on its inverse transcriptional relationship with the candidate gene signature, and subsequent molecular docking predicted potential interactions with SQLE and LSS. ML106 has previously been reported to exhibit biological activities involving targets such as tyrosinase and CLK4.^[28,29]^ However, there is currently no direct evidence demonstrating that ML106 modulates SQLE or LSS activity or exerts beneficial effects in sepsis. Importantly, neither transcriptional signature reversal nor molecular docking establishes biochemical target engagement or therapeutic efficacy. Therefore, the ML106 findings should be regarded as hypothesis-generating and primarily provide a basis for future biochemical, cellular, and in vivo validation.

This study has several limitations. First, the MIMIC-IV analysis was observational and adjusted only for age, sex, and BMI; therefore, residual confounding from comorbidities, inflammatory status, anemia-related factors, liver dysfunction, transfusion exposure, illness severity, and ICU-related characteristics cannot be excluded. The observed associations should not be interpreted as independent or causal effects. Second, the transcriptomic analysis involved mouse liver and cardiac tissues, and the observed overlap may partly reflect tissue- or context-specific responses. Although SQLE and RDH11 were supported in an independent human blood cohort, LSS was not, and bulk-blood expression may also be influenced by cell-composition differences. Third, the MR analysis was limited by the small number of genetic instruments for some genes, the relatively liberal LD threshold, and incomplete sensitivity analyses; thus, residual pleiotropy cannot be excluded, and the findings should be considered exploratory. Finally, the L1000FWD and molecular docking analyses were entirely computational. ML106 should therefore be regarded as a candidate drug for future investigation rather than a validated therapeutic agent, and further biochemical, cellular, and in vivo validation is required.

## 5. Conclusion

In conclusion, this integrative analysis identified nonlinear associations between circulating iron-related indices and sepsis status and revealed potential molecular links between iron deficiency and sepsis. Cross-tissue transcriptomic screening identified SQLE, LSS, and RDH11 as candidate genes, with external evaluation in human blood providing additional transcriptional support for SQLE and RDH11. Exploratory MR identified divergent genetically informed associations of SQLE and LSS expression with sepsis, although these findings require cautious interpretation and further validation. Exploratory drug prediction identified ML106 as a candidate drug for further investigation. Additional studies are required to determine whether the predicted interactions have biological or therapeutic relevance in sepsis.

## Author contributions

**Conceptualization:** Zehong Wu.

**Data curation:** Zehong Wu, Zhangqing Yi.

**Formal analysis:** Zehong Wu, Zhangqing Yi, Jiaming Wu.

**Funding acquisition:** Weizhi Zhang.

**Investigation:** Zehong Wu, Zhangqing Yi.

**Methodology:** Zehong Wu.

**Project administration:** Dongping Li, Weizhi Zhang.

**Resources:** Hanyi Yao, Dongping Li, Weizhi Zhang.

**Software:** Zehong Wu, Zhangqing Yi.

**Supervision:** Dongping Li, Weizhi Zhang.

**Validation:** Hanyi Yao, Dongping Li, Haojie Zhou, Weizhi Zhang.

**Visualization:** Zehong Wu.

**Writing – original draft:** Zehong Wu.

**Writing – review & editing:** Zhangqing Yi, Hanyi Yao, Dongping Li, Haojie Zhou, Jiaming Wu, Yuyang Huang, Weizhi Zhang.




