# Supplementary material for: Identification of potential key genes involved in iron deficiency for sepsis: A retrospective cohort and transcriptomic study
Source: Medicine (Baltimore). 2026 Sep 11;105(37):e50597. doi: 10.1097/MD.0000000000050597 (PMC13574409; doi:10.1097/MD.0000000000050597)
Supplement: Supplementary file 1 [file medi-105-e50597-s001.docx]

Table S1. Results of the MR-Egger intercept test for horizontal pleiotropy

| Gene | egger_intercept | egger_intercept_se | egger_intercept_pval |
| --- | --- | --- | --- |
| *SQLE* | -0.025 | 0.031 | 0.478 |
| *LSS* | -0.009 | 0.007 | 0.192 |
| *RDH11* | 0.004 | 0.025 | 0.909 |
