# Supplementary material for: Identification of potential key genes involved in iron deficiency for sepsis: A retrospective cohort and transcriptomic study
Source: Medicine (Baltimore). 2026 Sep 11;105(37):e50597. doi: 10.1097/MD.0000000000050597 (PMC13574409; doi:10.1097/MD.0000000000050597)
Supplement: Supplementary file 2 [file medi-105-e50597-s002.docx]

Table S2. Results of heterogeneity tests for Mendelian randomization analyses.

| Gene | method | Q | Q_df | Q_pval |
| --- | --- | --- | --- | --- |
| *SQLE* | MR Egger | 1.260 | 4 | 0.868 |
|  | Inverse variance weighted | 1.872 | 5 | 0.867 |
| *LSS* | MR Egger | 30.016 | 34 | 0.663 |
|  | Inverse variance weighted | 31.791 | 35 | 0.624 |
| *RDH11* | MR Egger | 0.054 | 1 | 0.816 |
|  | Inverse variance weighted | 0.074 | 2 | 0.963 |
